# Unmasking Nasal Septal Hematoma/Abscess: A Systematic Review and Meta‐analysis

**DOI:** 10.1002/oto2.174

**Published:** 2024-10-08

**Authors:** Douglas P. Nanu, Daniel Adelsberg, Shaun A. Nguyen, Nicholas P. Radulovich, Michele M. Carr

**Affiliations:** ^1^ Department of Otolaryngology–Head and Neck Surgery Charleston Medical University of South Carolina Charleston Washington USA; ^2^ Elson S. Floyd College of Medicine at Washington State University Spokane Washington USA; ^3^ Department of Otolaryngology Jacobs School of Medicine and Biomedical Sciences at the University of Buffalo Buffalo New York USA; ^4^ Department of Otolaryngology University at Buffalo Buffalo New York USA

**Keywords:** nasal septal abscess, nasal septal hematoma, saddle nose deformity

## Abstract

**Objective:**

We aim to discuss the demographics, symptoms, bacteriology, treatment, and sequelae associated with nasal septal hematoma/nasal septal abscess (NSH/NSA).

**Data Sources:**

CINAHL, PubMed, and Scopus were searched from inception until October 15, 2023.

**Review Methods:**

Preferred Reporting Items for Systematic Reviews and Meta‐analysis 2020 guidelines were followed. Inclusion criteria included patients who were diagnosed with a traumatic NSH/NSA. NSH/NSA due to surgical procedures was excluded. Demographics included N of patients, patient age, and gender. Symptoms, antibiotics given, bacteriology, and sequelae were analyzed. Meta‐analysis of continuous measures (mean, median), and proportions (%) with a 95% confidence interval (CI) was conducted.

**Results:**

Thirty studies (N = 598) were included. In total, 72.1% were males (95% CI: 67‐78). The total mean age was 21.6 years (range: 0.2‐85, 95% CI: 17.2‐26.1). The mean time from trauma to diagnosis was 8.2 days. Common symptoms at presentation included nasal obstruction/congestion at 60.3% (95% CI: 37.1‐81.4), nasal pain at 30.0% (17.2‐44.6), swelling at 20.4% (8.7‐35.5), headache at 15.5% (7.3‐26.0), and fever at 13.9% (7.3‐22.2). The most common pathogens isolated included *Staphylococcus* *aureus* at 56.5% (49.0‐63.8), *Streptococcus* species at 8.9% (5.2‐14.0), and *Klebsiella pneumoniae* at 6.3% (3.2‐10.8). Antibiotics given included amoxicillin‐clavulanate at 10.3% (4.5‐18.2), metronidazole at 9.5% (1.1‐24.9), ampicillin‐sulbactam at 8.9% (0.4‐26.5), and unspecified antibiotics at 39.7% (13.8‐69.2). The most common sequelae were nasal septal deformity/cartilage destruction at 14.3% (7.7‐22.6).

**Conclusion:**

NSA/NSH has an 8‐day delay in diagnosis from the time of trauma. First‐line practitioners should be made aware of the signs and symptoms of this condition to minimize the risk of morbidity.

Nasal injuries are the most common facial injuries, and while most cases do not require immediate attention, some may require prompt medical intervention for proper assessment and management. One diagnosis that requires prompt intervention is nasal septal hematoma (NSH) which has a high chance of morbidity if missed, misdiagnosed, or mismanaged.^1^ NSH most commonly occurs due to trauma. Less commonly, it manifests due to sinusitis, influenza, dental infections, and iatrogenic causes.^2^, ^3^, ^4^ There have been previous reports of spontaneous NSH with idiopathic causes often associated with immunodeficiency.^5^, ^6^ The pathophysiology of NSH is attributed to the accumulation of blood under the mucoperichondrium or mucoperiosteum of the septal cartilage or bone. This accumulation of blood deprives the septal cartilage of its blood supply subsequently causing cartilage ischemia and eventual necrosis.^7^ It is currently estimated that NSH occurs in 0.8% to 1.6% of patients with a nasal injury, however, the exact incidence is unknown.^1^ The most common symptoms of NSH include nasal obstruction, pain, rhinorrhea, and fever; however other symptoms have also been reported.^1^ Local complications include nasal septal abscess (NSA), which can lead to a nasal septal perforation, saddle nose deformity secondary to perforation, deviated nasal septum, facial cellulitis, or nasal vestibulitis.^1^ Systemic complications include bacteremia which can lead to sepsis, posing a significant risk of mortality.^8^ It may be difficult to distinguish clinically between an NSH and an NSA, especially if a longer period has elapsed following nasal injury or symptom presentation.^9^, ^10^ NSH treatment encompasses incision and drainage (I&D) coupled with nasal packing. In cases where there is suspicion for infection, a wound culture should be performed, accompanied by sensitivity testing. Patients are prescribed a targeted treatment approach, typically involving the administration of trimethoprim‐sulfamethoxazole or doxycycline as *Staphylococcus aureus* is the most common pathogen found in NSA; however, the choice of antibiotics will differ based on the culture and sensitivity results.^7^

A 2011 review focused on time to diagnosis, management, and prevention of NSH/NSA; however, it was not a systematic review, and meta‐analysis was not conducted.^11^ This review will be the first systematic review with meta‐analysis which aims to enhance our understanding of NSH/NSA by analyzing demographic variables, clinical, treatment modalities, antibiotics given, culture results, comorbidities, and imaging done. Additionally, patient follow‐up times and time from trauma to diagnosis were also analyzed.

## Methods

### Search Strategy

This study was conducted according to Preferred Reporting Items for Systematic Reviews and Meta‐analysis guidelines.^12^ Two researchers (D.P.N. and D.A.) independently performed a literature search to identify potentially relevant studies via Scopus, PubMed, CINAHL, and Cochrane libraries. For any conflicts after discussion, author S.A.N. made the final decision and resolved conflicts. The downloaded full‐text articles for the final studies were then stored.

A search was performed from inception until October 15, 2023, in each database. Keywords and Phrases included: “Nasal septal hematoma,” “Nasal septal haematoma,” “Nasal septal hematomas,” “Nasal septal haematomas,” “Nasal septal abscess,” OR NSH.

### Study Selection

For a study to be included, (1) the article had to include subjects who were diagnosed with an NSH or NSA, and (2) the article had to state how many patients were included. We excluded (1) studies that were not written in English, (2) studies where patients acquired an NSH/NSA following surgery (eg, rhinoplasty, septoplasty), and (3) studies that were case reports, discussions, editorials, book chapters, commentaries, or systematic reviews with or without meta‐analysis.

### Data Extraction

Data collected from each study was extracted into a spreadsheet (Excel 2023; Microsoft Corporation). Extracted data included ordinal data such as the total number of patients, nominal data such as gender (males and females), symptoms, treatments, antibiotics used, sequelae, culture results, comorbidities, and imaging used. Continuous data extracted included age, follow‐up time, and time from trauma to diagnosis. Articles were reviewed twice independently by the 2 researchers (D.P.N. and D.A.) to verify the proper extraction of data and extracted independently.

### Methodological Quality of Included Studies

**Figure 1:**
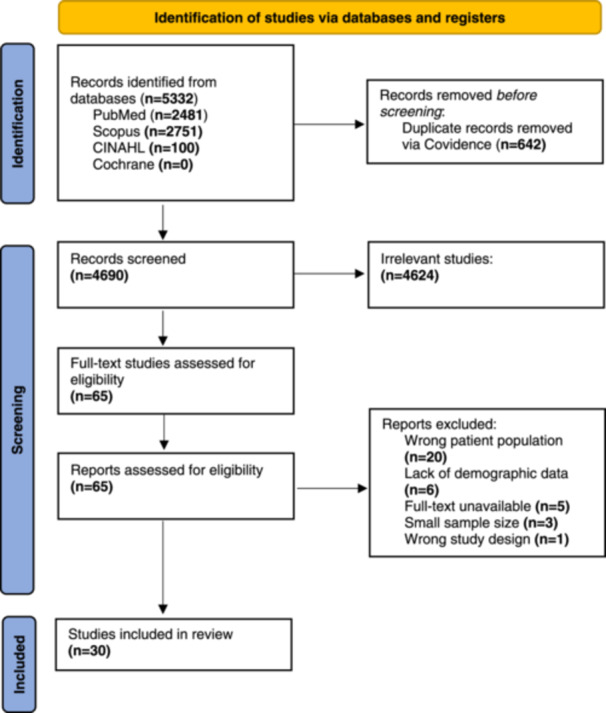
Preferred Reporting Items for Systematic Reviews and Meta‐analysis 2020 flow diagram for new systematic reviews which included searches of databases and registers only.

Studies included 27 case series, 2 retrospective cohort studies, and 1 case‐control study. We screened 5332 studies for potential relevance via Covidence (Covidence systematic review software, Veritas Health Innovation, Melbourne, Australia); 65 nonduplicated full‐text studies were assessed for eligibility (Figure 1). English studies were included. After reviewing 65 full‐text studies, 35 English language studies were included in the analysis.

The level of evidence for selected articles was assessed using the Oxford Center for Evidence‐Based Medicine criteria.^13^ The Risk Of Bias In Nonrandomized Studies‐of Interventions (ROBINS‐I) tool was used to assess the risk of bias in all retrospective cohort and case‐control studies.^14^ The risk of bias items for these nonrandomized studies included confounding, selection of participants into the study, classification of interventions, deviations from intended interventions, missing data, measurement of outcomes, and selection of the reported result. Each aspect of the risk of bias was assigned a grade of low, unclear, or high. Two authors (D.P.N and N.P.R.) performed a pilot assessment on 3 studies to check for consistency of risk of bias assessment. Both authors then performed an independent assessment of the risk of bias in the remaining studies. The Joanna Briggs Institute (JBI) Critical Appraisal Checklist for Case Series was used to assess case series risk of bias. Both authors (D.P.N. and N.P.R.) independently reviewed the articles using the checklists and rated each item as “yes,” “no,” “unclear,” or “not applicable.” Each item was given a score of “1” for “yes” and “0” for “no,” “not applicable,” or “unclear.” The case series checklists were scored out of 10. A score of 5 or higher on either checklist was considered at low risk for bias and was, therefore, included in the paper (Table 1). All risk of bias disagreements were resolved by the third author (S.A.N).

**Table 1:** JBI Critical Appraisal Tool Case Series

Study	1	2	3	4	5	6	7	8	9	10	Overall
Ali et al^15^	Y	Y	Y	Y	Y	Y	Y	Y	Y	Y	10, include
Alvarez et al^16^	Y	Y	Y	Y	Y	Y	Y	Y	Y	Y	10, include
Ambrus et al^2^	Y	Y	Y	Y	Y	N	Y	Y	Y	Y	9, include
Brook^17^	N	Y	Y	U	U	Y	Y	Y	N	Y	6, include
Canty and Berkowitz^18^	Y	Y	Y	Y	Y	Y	Y	Y	Y	Y	10, include
Cheng et al^19^	Y	Y	Y	Y	Y	Y	Y	Y	Y	Y	10, include
Chukuezi^20^	Y	Y	Y	U	Y	Y	Y	Y	Y	Y	9, include
Close and Guinness^21^	N	Y	Y	U	Y	Y	Y	Y	Y	Y	8, include
da Silva et al^3^	N	Y	Y	U	U	Y	Y	Y	Y	Y	7, include
Debnam et al^22^	Y	Y	Y	U	U	Y	Y	Y	Y	Y	8, include
Dinesh et al^23^	N	Y	Y	U	U	Y	Y	Y	Y	Y	7, include
Dispenza et al^24^	N	Y	Y	U	U	Y	Y	Y	Y	Y	7, include
Elcock^25^	N	Y	Y	U	Y	Y	Y	Y	Y	Y	8, include
Ghadersohi et al^26^	Y	Y	Y	U	N	Y	Y	Y	Y	Y	8, include
Jalaludin^27^	Y	Y	Y	Y	Y	Y	Y	Y	Y	Y	10, include
Kryger and Dommerby^28^	Y	Y	Y	Y	Y	N	Y	Y	Y	Y	9, include
Menger et al^29^	Y	Y	Y	Y	Y	Y	Y	Y	Y	Y	10, include
Nwosu and Nnadede^30^	Y	Y	Y	Y	Y	N	Y	Y	Y	Y	9, include
Sandel 4th and Davison^5^	Y	Y	Y	Y	Y	Y	Y	Y	N	Y	9, include
Sayin et al^31^	Y	Y	Y	Y	N	Y	Y	Y	Y	Y	9, include
Sayin et al^32^	Y	Y	Y	Y	N	Y	Y	Y	Y	Y	9, include
Shah et al^6^	Y	Y	Y	Y	Y	Y	Y	Y	Y	Y	10, include
Shapiro^33^	N	Y	Y	U	U	Y	Y	Y	Y	Y	7, include
Sogebi and Oyewole^34^	Y	Y	Y	U	N	Y	Y	Y	Y	Y	8, include
Tavares et al^35^	Y	Y	Y	U	U	N	U	U	Y	Y	5, include
Tien et al^4^	Y	Y	Y	Y	Y	Y	Y	U	Y	Y	9, include
Wasilewska and Zawadzka‐Głos^36^	N	Y	Y	U	U	U	Y	Y	U	Y	5, include

Abbreviations: JBI, Joanna Briggs Institute; N, no; U, unclear; Y, yes.

1. Were there clear criteria for inclusion in the case series?

2. Was the condition measured in a standard, reliable way for all participants included in the case series?

3. Were valid methods used for identification of the condition for all participants included in the case series?

4. Did the case series have consecutive inclusion of participants?

5. Did the case series have complete inclusion of participants?

6. Was there clear reporting of the demographics of the participants in the study?

7. Was there clear reporting of clinical information of the participants?

8. Were the outcomes or follow up results of cases clearly reported?

9. Was there clear reporting of the presenting site(s)/clinic(s) demographic information?

10. Was statistical analysis appropriate?

### Statistical Analysis

Meta‐analysis of continuous measures (age, follow‐up time, etc,) was performed with Cochrane Review Manager version 5.4 (The Cochrane Collaboration, 2020). Meta‐analysis of proportions was performed using MedCalc 20.305 (MedCalc Software). Each measure (mean/proportion [%] and 95% confidence interval [CI]) was weighted according to the number of patients affected. As some studies reported the outcomes in the median (first quartile, third quartile), the quantile estimation method was deployed to calculate the pooled estimates.^37^, ^38^ Heterogeneity among studies was assessed using *χ* ^2^ and *I* ^2^ statistics with fixed effects (*I* ^2^ < 50%) and random effects (*I* ^2^ > 50%).

Finally, potential publication bias was evaluated by visual inspection of the funnel plot and Egger's regression test, which statistically examines the asymmetry of the funnel plot.^39^, ^40^ A *P* < .05 was considered to indicate a significant difference for all statistical tests.

## Results

**Figure 2:**
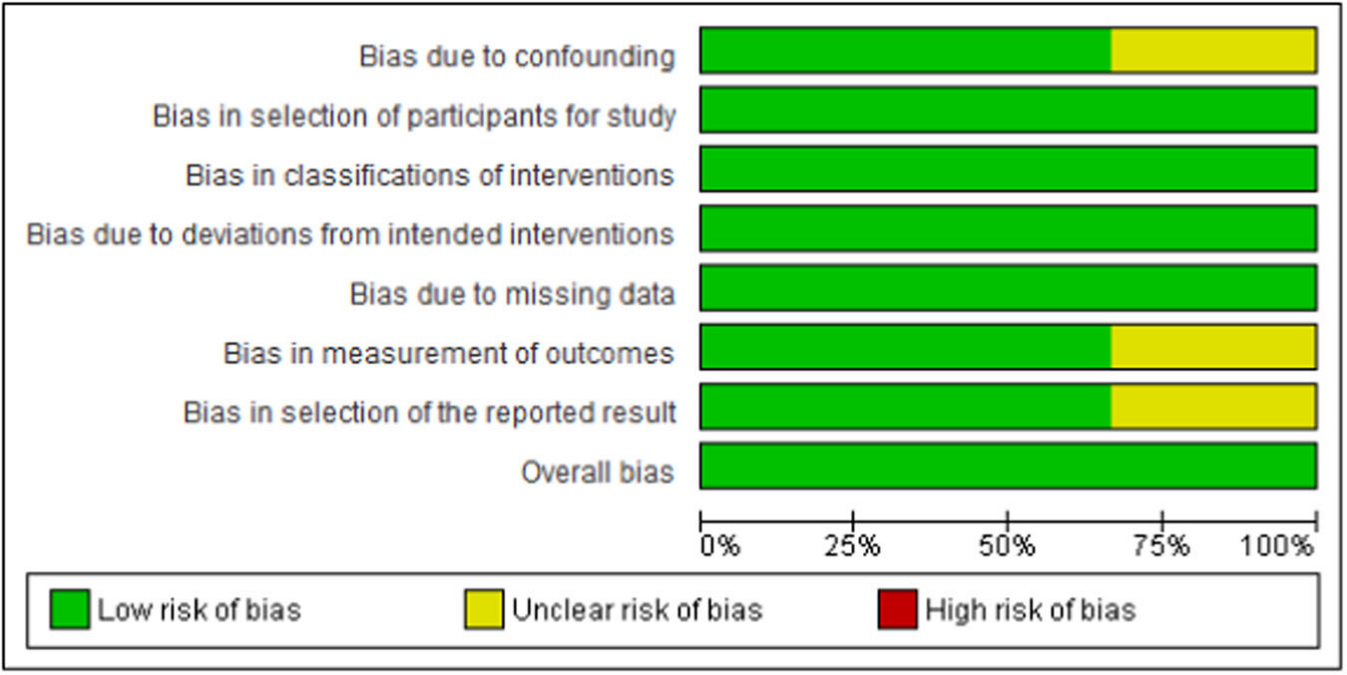
Risk of bias graph: review authors' judgements about each risk of bias item presented as percentages across all included studies.

**Figure 3:**
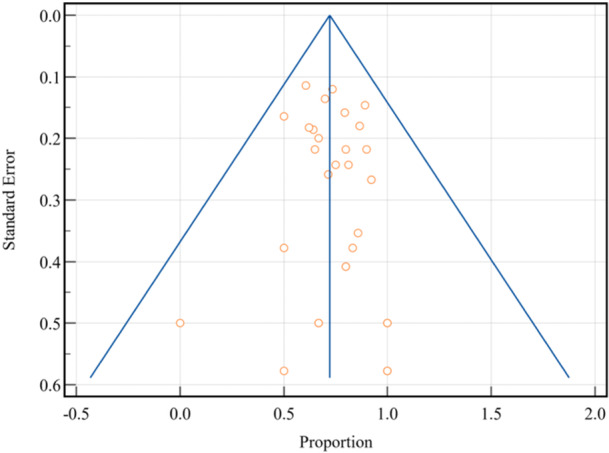
Egger's test for publication bias.

Out of 4690 studies screened, 30 studies (N = 598) were included. Data from 598 patients diagnosed with an NSH/NSA were extracted. Studies included for analysis were published from 1978 to 2023 and originated from 14 different countries. Most of the studies included in this review were Oxford Level of Evidence 4 except for 3 studies which were Level 3. Critical appraisal of nonrandomized studies (Figure 2) indicated an overall acceptably low risk of bias with potential sources of bias being most pronounced due to bias due to confounding, bias in the measurement of outcomes, and bias in the selection of the reported result. JBI appraisal of the 27‐case series (5‐10 scores) found all to have good quality and low risk of publication bias (Table 1). A funnel plot (Figure 3) with Egger's test (0.256, 95% CI: −0.892 to 0.140, *P* = .651) suggested little publication bias, as all the studies were within the funnel with little asymmetry.

All studies analyzed contained patient biological sex data. Out of 598 patients, 72.1% (95% CI: 68.4‐75.6) were males and 27.9% (95% CI: 24.4‐31.6) were females. The mean patient age was 21.6 years (range: 0.2‐85, 95% CI: 17.2‐26.1). The mean time from trauma to diagnosis was 8.2 days (range: 0.08‐28, 95% CI: 6.2‐10.2). The mean time to follow up was 30.0 months (range: 0.8‐111, 95% CI: 14.5‐45.4).

There were notable studies with cases where patients did not acquire an NSH/NSA due to trauma. The total number of nontraumatic etiology and possible etiologies per study can be found in Table 2.

**Table 2:** Studies With Nontraumatic Etiologies

Study	Total patients (N)	Nontraumatic/unknown etiology (N)	Etiology (N)
Ambrus et al^2^	16	3	Unknown (3)
Dinesh et al^18^	3	3	DM (3)
Cheng et al^17^	6	4	DM (3) Acute sinusitis (1)
Jalaludin^19^	14	2	Chronic sinusitis (1) Vestibulitis with uncontrolled DM (1)
Kryger and Dommerby^20^	52	1	Upper respiratory infection (1)
Ngo et al^30^	36	17	DM (17)
Nwosu and Nnadede^21^	53	15	Unknown (14) Tobacco snuffing (1)
Sandel 4th and Davison^5^	3	3	HIV (3)
Shah et al^6^	3	3	Nasal furunculosis (1) Intranasal drug abuse (1) Sphenoid sinusitis (1)
Shapiro^23^	3	1	Unknown (1)
Sogebi and Oyewole^24^	31	5	Unknown (5)
Tavares et al^25^	30	5	Vestibulitis (3)Unknown (2)
Tien et al^4^	5	4	Unknown (2)Acute rhinosinusitis (2)

Abbreviations: DM, diabetes mellitus; HIV, human immunodeficiency virus.

The 3 most common symptoms included nasal obstruction/congestion in 60.3% (95% CI: 37.1‐81.4), nasal pain in 30.0% (95% CI: 17.2‐44.6) and nasal swelling in 20.4% (95% CI: 8.7‐35.5) of the patients. Additional symptoms can be found in Table 3.

**Table 3:** NSH/NSA Symptom(s)

Symptom(s)	Proportion (%)	95% CI
Nasal obstruction/congestion	60.3	37.1‐81.4
Nasal pain	30.0	17.2‐44.6
Nasal swelling	20.4	8.7‐35.5
Headache	15.5	7.3‐26.0
Fever	13.9	7.3‐22.2
Hyperemia of nasal mucosa	6.5	0.4‐19.0
Mouth breathing	5.7	0.6‐15.7
Rhinorrhea	5.5	2.1‐10.3
Epistaxis/ecchymosis	5.5	2.2‐10.2

Abbreviations: CI, confidence interval; NSA, nasal septal abscess; NSH, nasal septal hematoma.

Positive nasal septum culture results were found in 163 patients. The most common pathogen was *S. aureus* in 56.5% (95% CI: 49.0‐63.8), *Streptococcus* species in 8.9% (95% CI: 5.2‐14.0), *Klebsiella pneumoniae* in 6.3% (95% CI: 3.2‐10.8), *P. aeruginosa* in 6.2% (95% CI: 3.2‐10.8), *S. pneumoniae* in 5.3% (95% CI: 2.5‐9.6), and coliforms in 3.1% (95% CI: 1.1‐6.8) of patients who had nasal septum cultures taken.

There were antibiotics given for this condition. However, unspecified antibiotics were the most common, given to 39.7% (95% CI: 13.8‐69.2) of patients. The most notable specific antibiotics given were amoxicillin‐clavulanate at 10.3% (95% CI: 4.5‐18.2), metronidazole at 9.5% (95% CI: 1.1 ‐ 24.9), ampicillin‐sulbactam at 8.9% (95% CI: 0.4‐26.5), and ceftazidime at 4.7% (95% CI: 0.7‐12.0) of patients. Additional antibiotic treatments can be found in Table 4.

**Table 4:** NSH/NSA Antibiotic Treatment(s)

Antibiotic treatment(s)	Proportion (%)	95% CI
Unspecified antibiotics	39.7	13.8‐69.2
Amoxicillin clavulanate	10.3	4.5‐18.2
Metronidazole	9.5	1.1‐24.9
Ampicillin‐sulbactam	8.9	0.4‐26.5
Ceftazidime	4.7	0.7‐12.0
Floxacillin	4.4	1.3‐9.3
Ceftriaxone	3.2	1.8‐5.4
Amoxicillin	1.8	0.7‐3.5
Penicillin	1.7	0.7‐3.4
Oxacillin	1.7	0.7‐3.4
Ampicillin	1.3	0.5‐2.9
Trimethoprim‐sulfamethoxazole	1.3	0.4‐2.5

Abbreviations: CI, confidence interval; NSA, nasal septal abscess; NSH, nasal septal hematoma.

Sequelae included rhinoseptal deformity/cartilage destruction (saddle nose deformity/septal perforation) in 14.3% (95% CI: 7.7‐22.6) of patients. The most common comorbidity was diabetes mellitus (DM) in 4.8% (95% CI: 2.0 ‐ 8.7) followed by hypertension in 2.1% (95% CI: 1.1‐3.5) of patients.

The most common method of imaging used was a computerized tomography scan in 15.2% (95% CI: 5.3‐28.9) of patients, followed by a plain film X‐ray in 1.4% (95% CI: 0.7‐2.7).

## Discussion

### Demographics

In our study, the mean patient age was 21.6 years suggesting that younger patients are more frequently affected by NSH. 72.1% of patients in our study were males. Previous research suggests that males have a higher risk of facial trauma than females during the first 70 years of life.^42^, ^43^

In a previous study of children diagnosed with an NSH/NSA, 92% of the patients were male. Trauma was identified as the primary cause of an NSH/NSA in 85% of all the cases in this study.^15^ Additionally, a previous review article also mentioned that the majority of NSH/NSA patients diagnosed were male children.^11^

Our mean time to diagnosis was 8.2 days, similar to the median time to diagnosis of 7.0 days previously seen in the literature.^15^ Time to diagnosis in NSH/NSA abscess is crucial because if not treated promptly the disease process can rapidly progress and lead to severe complications such as tissue necrosis and septal perforation. NSH/NSA can cause significant nasal obstruction. This can lead to respiratory distress, especially in those who already have respiratory disease. Permanent damage to the nasal septum can result in cosmetic problems. A nasal abscess that is not treated promptly can lead to sepsis.^1^

### Nontraumatic/Spontaneous Etiology

Instances of spontaneous NSH and cases with unknown etiology have been documented in the literature. In our review, there were 13 studies (11.0%, N = 66) that highlighted this.^2^, ^4^, ^5^, ^6^, ^19^, ^23^, ^27^, ^28^, ^30^, ^33^, ^34^, ^40^, ^41^ The most prevalent etiology claimed in the articles was DM. Among cases unrelated to trauma, 24 patients with DM were diagnosed with an NSA/NSH without other related antecedent events usually associated with NSA/NSH. DM can impact the vascular system, which is exacerbated when DM is uncontrolled. Excessive periods of hyperglycemia decrease the elasticity of blood vessels, causing subsequent narrowing which impedes blood flow. DM can lead to complications in the vessels of the nasal septum making them more susceptible to damage and thus increasing the likelihood of hematoma formation.^44^, ^45^ After septoplasty, the risk of NSA was increased in patients with higher DM severity which was measured by the Diabetes Complications Severity Index (DCSI). The hazard ratio (HR) for NSA incidence in patients with type 2 DM was 2.62. For patients with a DCSI greater than or equal to 1, the HR for NSA risk was even higher showing greater than a 3.5× increased risk. This is consistent with the findings in this review. Diabetic patients have an increased susceptibility to infection when presenting with an NSH.^46^

Sinusitis was another notable etiology of NSA. Five patients in the study presented with NSA attributed to this cause: 3 were associated with acute rhinosinusitis (ARS),^4^, ^19^ 1 with chronic rhinosinusitis,^27^ and 1 with sphenoid sinusitis.^6^ In a case series, NSA was found in 2 patients with ARS involving the bilateral frontal, maxillary, and ethmoid sinuses with one of these patients having an ipsilateral middle turbinate concha bullosa.^4^

### Symptoms

In many cases, symptoms of an NSH/NSA are nonspecific.^1^ The most common symptom seen was nasal obstruction and congestion in 60.3% (95% CI: 37.1‐81.4) of patients. According to the literature, this is not surprising, since nasal obstruction and congestion can be seen in up to 95% of patients with an NSH.^1^ The second most common symptom included nasal pain which was seen in 30.0% (95% CI: 17.2‐44.6) of patients. Previous literature stated that nasal pain was experienced in 50% of patients however a CI was not given therefore we do not know the possible variability of this value.^1^ The next most common symptom was nasal swelling in 20.4% of patients (95% CI: 8.7‐35.5). This percentage is lower than what was reported in a previous case series in children with NSH/NSA due to trauma, where 43.0% of patients exhibited swelling or ecchymosis.^16^

### Pathogens

Out of the 163 patients who had a positive nasal culture, the most common pathogen found in this review was *S. aureus.* Previous reports indicate that *S. aureus* was present in 70% of all nasal septal abscesses.^11^ The next most common pathogen found was from the *Streptococcus* genus which made up 8.9% (95% CI: 5.2‐14.0) of the cases. Despite yielding positive results for this genus, studies in this review did not specify the particular species within it. Lastly, *K*. *pneumoniae* was seen in 6.3% (95% CI: 3.2‐10.8) of patients. This is consistent with the literature for NSA stating that *K. pneumoniae* along with other *Enterobacteriaceae* are less commonly found.^11^

### Treatments

All patients in this review (N = 598) were treated with I&D. This procedure is performed to evacuate the accumulated blood and prevent complications such as cartilage necrosis and absorption if not promptly or properly managed.^47^ Most of the studies did not specify the antibiotics used for treatment. One study (N = 53) within this category mentioned that “broad antibiotics” were given to all patients.^30^ The next most prescribed antibiotic was amoxicillin‐clavulanate, given to 10.3% (95% CI: 4.5‐18.2) of patients. Amoxicillin‐clavulanate is effective for treating infections caused by *S. aureus*, specifically methicillin‐sensitive *S. aureus*.^48^ Additionally, this antibiotic has been shown to be active against most strains of *Streptococcus* and *Klebsiella* species which were both also identified in this review.^48^ Following amoxicillin‐clavulanate, metronidazole was given the most often administered in 9.5% (95% CI: 1.1‐24.9) of patients. Sayin et al reported that metronidazole along with amoxicillin‐clavulanate was given for septal abscess in 36 patients.^31^ Ngo et al^41^ reported on 7 patients who initially received amoxicillin‐clavulanate with metronidazole, while 29 others got ceftazidime with metronidazole. Among those on amoxicillin‐clavulanate plus metronidazole, 4 patients (57.14%) were switched to ciprofloxacin or ceftazidime combined with metronidazole based upon receiving antibiogram results showing high patterns of resistance to penicillin and oxacillin combined with low levels of resistance to ciprofloxacin or ceftazidime.^41^ In 38.89% of cases the infection remained severe or active after 2 to 3 days, and culture results showed bacterial resistance to the initial treatment, so the antibiotic was changed. Ciprofloxacin was the most common alternative (30.56%), followed by vancomycin and ceftazidime (5.56% and 2.27%, respectively).^41^ Lastly, in a series of 3 patients, 2 patients were given metronidazole along with cefuroxime.^23^ As evidenced for all patients that were given metronidazole, this antibiotic was always given along with another type of antibiotic and never as the sole therapy. However, none of the studies explicitly mentioned why metronidazole was added, and for what specific pathogen. Metronidazole is mostly used to treat infections caused by various anaerobic bacteria and protozoa, including *Trichomonas vaginalis, Heliobacter pylori* and certain specific of *Bacteroides, Clostridium, Peptococcus, Peptostreptococcus*, and *Prevotella* among others.^49^ None of the studies in which metronidazole was given mentioned infection with these pathogens.

### Limitations

This review has some limitations that are worthy of discussion. First, this is a systematic review with meta‐analysis using mostly case series which have several limitations on their own. Case series lack generalizability since they often involve a small number of patients. Since these studies are observational, they cannot definitively establish a cause‐effect relationship between an exposure and an outcome. There is also the danger of overinterpretation of the findings as the results are based only on a small number of observations. Case series may be subject to publication bias, as studies with positive or novel findings are more likely to be published. Lastly, there are inconsistencies in reporting, especially between different journals. This results in variation of the level of detail provided, making it difficult to evaluate outcomes in a consistent manner across different studies. This inconsistency in reporting can hinder the synthesis of evidence in a systematic review with meta‐analysis.^50^

## Conclusion

Younger male patients presenting with a facial injury warrant a high index of suspicion for an NSH/NSA. Although *S. aureus* is the most common pathogen found, other pathogens may also be involved, underscoring the importance of culturing NSA before treatment. Notably, NSA/NSH has an 8‐day delay in diagnosis from the time of trauma. First‐line practitioners should be made aware of the signs and symptoms of NSH because permanent sequelae are possible. Lastly, other comorbid conditions such as DM and sinusitis may increase the odds of NSH/NSA unrelated to trauma, however, more studies need to be performed to see if such an association exists.

## Author Contributions

**Douglas P. Nanu**, substantial contributions to the conception or design of the work and the acquisition, analysis, and interpretation of data for the work, drafting the work and reviewing it critically for important intellectual content, final approval of the version to be published and agreement to be accountable for all aspects of the work in ensuring that questions related to the accuracy or integrity of any part of the work are appropriately investigated and resolved; **Daniel Adelsberg**, substantial contributions to the conception or design of the work and the acquisition, analysis, and interpretation of data for the work, drafting the work and reviewing it critically for important intellectual content, final approval of the version to be published and agreement to be accountable for all aspects of the work in ensuring that questions related to the accuracy or integrity of any part of the work are appropriately investigated and resolved; **Shaun A. Nguyen**, substantial contributions to the conception or design of the work, analysis, and interpretation of data for the work, drafting the work and reviewing it critically for important intellectual content, final approval of the version to be published and agreement to be accountable for all aspects of the work in ensuring that questions related to the accuracy or integrity of any part of the work are appropriately investigated and resolved; **Nicholas P. Radulovich**, substantial contributions to the conception or design of the work and the acquisition, analysis, and interpretation of data for the work, drafting the work and reviewing it critically for important intellectual content, final approval of the version to be published and agreement to be accountable for all aspects of the work in ensuring that questions related to the accuracy or integrity of any part of the work are appropriately investigated and resolved; **Michele M. Carr**, substantial contributions to the conception or design of the work and the acquisition, analysis, and interpretation of data for the work, drafting the work and reviewing it critically for important intellectual content, final approval of the version to be published and agreement to be accountable for all aspects of the work in ensuring that questions related to the accuracy or integrity of any part of the work are appropriately investigated and resolved.

## Disclosures

### Competing interests

All authors (Douglas P. Nanu, Daniel Adelsberg, Shaun A. Nguyen, Nicholas P. Radulovich, Michele M. Carr) report no financial support or funding.

### Funding source

All authors (Douglas P. Nanu, Daniel Adelsberg, Shaun A. Nguyen, Nicholas P. Radulovich, Michele M. Carr) report no conflicts of interest to disclose.
